# Sodium-Glucose Cotransporter-2 (SGLT2) Inhibitors and Cardiovascular Outcomes: A Review of Literature

**DOI:** 10.7759/cureus.63796

**Published:** 2024-07-04

**Authors:** Sweatha Mani, Abirami Balasubramanian, Keerthana Veluswami, Sudipta Rao, Shailesh Aggarwal

**Affiliations:** 1 Internal Medicine, K.A.P. Viswanatham Government Medical College, Tiruchirappalli, IND; 2 Internal Medicine, Stanley Medical College and Hospital, Chennai, IND; 3 Internal Medicine, JSS Medical College, Mysore, IND

**Keywords:** empa-reg outcome trial, canvas program, declare-timi 58 trial, cardiovascular outcome trials, gliflozins, sodium-glucose cotransporter 2 inhibitor

## Abstract

Coronary arterial diseases are a major contributor to disease and death worldwide and are most often compounded by several other underlying medical conditions. A key concern is type 2 diabetes mellitus (T2DM). Despite progress in medical advancements, these life-threatening illnesses are still underdiagnosed and undermanaged. A relatively newer class of anti-diabetic drugs, the sodium-glucose cotransporter-2 inhibitors (SGL2-Is), also termed gliflozins, have shown promising results in reducing cardiovascular risk, regardless of diabetic status. These drugs have on-target (promoting renal glycosuria and diuresis by acting on the SGLT-2 channels in the proximal convoluted tubule) and off-target effects contributing to the reported cardiovascular benefit. Some emerging theories about its impact on myocardial energetics, calcium balance, and renal physiology exist. In this review article, we explored three major cardiovascular outcome trials: the Dapagliflozin Effect on Cardiovascular Events-Thrombolysis in Myocardial Infarction 58 (DECLARE-TIMI 58) trial, the CANagliflozin cardioVascular Assessment Study (CANVAS) program, and the Empagliflozin Cardiovascular Outcome Event Trial in Type 2 Diabetes Mellitus Patients-Removing Excess Glucose (EMPA-REG OUTCOME) trial to evaluate the cardiovascular effects of SGLT2-Is.

## Introduction and background

Diabetes mellitus (DM) is a lasting condition impacting metabolism, either due to defective insulin secretion or peripheral organ resistance to insulin, characterized by elevated blood sugar levels. Long-term effects include organ dysfunction and damage, especially to the eyes, kidneys, nerves, heart, and blood vessels [1]. The Diabetes Atlas from the International Diabetes Federation (IDF) showed a prevalence rate of 8.6% in adults and an estimated 425 million people with DM [2]. Diabetes mellitus is complicated by a large number of cardiovascular diseases, which increase morbidity and mortality significantly [3, 4]. Having type 2 diabetes mellitus (T2DM) doubles the likelihood of cardiovascular disease [5]. With the introduction of drugs to reduce hospitalization due to heart failure (HHF) and to extend survival, there have been significant advancements in the treatment of heart failure (HF) patients with reduced ejection fraction over the last few decades [6]. Gliflozins, or sodium-glucose cotransporter-2 inhibitors (SGLT2-Is), are a subset of anti-diabetic drugs that act by inhibiting the SGLT2 channels in the renal proximal convoluted tubule, which reabsorbs about 90% of the filtered glucose [7]. They reduce the renal threshold for glucose excretion from 180 mg/dl to 40 mg/dl, lowering blood glucose levels [8]. In the heart, these drugs modulate the sodium-hydrogen (Na-H) exchangers [9] and also exert beneficial effects on isolated cardiomyocytes [10, 11]. The proposed mechanisms through which they exert cardioprotective effects are lowering blood pressure, improving natriuresis, preventing inflammation and detrimental cardiac remodeling, preventing reperfusion injury, acceleration of autophagy and lysosomal degradation, increasing erythropoietin (EPO) levels and circulating pro-vascular progenitor cells, and decreasing epicardial fat mass and oxidative stress [12-16]. It is estimated that among two million eligible patients (69% of total HF patients) in the United States, incorporating an SGLT2 inhibitor may prevent or delay up to 34,125 deaths per year [17]. The primary aim of this research is to focus on the similarities and differences between the various cardiovascular outcome trials (CVOTs) conducted and to emphasize the importance of SGLT2-Is in improving cardiovascular health in detail.

## Review

Methodology

Search Strategy

A comprehensive literature search was conducted to identify articles related to SGLT2-Is and cardiovascular outcomes. We searched across databases such as PubMed and Google Scholar. The following keywords were used to search the articles: "sodium-glucose cotransporter 2 inhibitors", "gliflozins", "cardiovascular outcome trials", "DECLARE-TIMI 58 trial", "CANVAS program", "EMPA-REG outcome trial", "major adverse cardiovascular events", and "hospitalization due to heart failure".

Selection Criteria

All kinds of studies conducted across nations have been included in the paper. Studies on patients with T2DM, with or without atherosclerotic cardiovascular disease, were selected for the study. Studies conducted in the last 10 years were reviewed. Unpublished works were not included.

Ethical Considerations

As this is a review article, no ethical approval was required. However, ethical considerations in the original studies were taken into account while interpreting the findings.

Physiology of SGLT2 cotransporters in the kidney

The two main SGLT2 cotransporters are SGLT1, found in the small intestine, heart, skeletal muscle, and kidney, and SGLT2, exclusively in the kidneys [18-20]. SGLT2 cotransporters are found in the brush border of the S1 and S2 regions of the proximal convoluted tubule (PCT) and reabsorb up to 97% of filtered glucose as they have a high transport capacity for glucose. Since they have low transport capacity, SGLT1 cotransporters in the S3 segment absorb the remaining 3%-10% of glucose. This synergy between the SGLT cotransporters handles the full load of filtered glucose. This reabsorption reaches the threshold limit (Tmax) when the glucose levels in the blood are between 180 and 200 mg/dl. Past this limit, glucosuria occurs [8]. However, in DM, to manage the rise in glucose flow in the lumen, the kidneys shift the Tmax levels to 240 mg/dl [8], further boosting the amount of SGLT2 cotransporters [21]. This causes energy consumption by the Na-K-ATPase at the basolateral membrane, which plays a pivotal role in the development of kidney problems in diabetic individuals [22].

Mechanism of action of SGLT2-Is

Phlorizin, derived from apple tree root bark, is the first SGLT2 inhibitor and a naturally occurring phenolic glycoside. When administered to dogs, it causes glucosuria, polyuria, and weight loss, which is a diabetic-like state [23]. Phlorizin acts only when given through the intravenous route. To combat this, T-1095 was designed to be given orally. It worked on both SGLT1, causing adverse gastrointestinal side effects and intolerance, and SGLT2, causing microalbuminuria and weight loss in rats [24,25]. Subsequently, seven orally-acting drugs have been designed, exhibiting a higher affinity for SGLT2 inhibition than SGLT1 [26].

By promoting urinary sodium and glucose elimination, which reduces the plasma and interstitial fluid volumes, gliflozins bring down the preload on the heart, thereby contributing to cardiac remodeling [27]. SGLT2 inhibition promotes natriuresis [28] and reduced plasma volume, evident from a drop in systolic blood pressure by 3-6 mmHg and diastolic by 1-1.5 mmHg [29, 30]. Hyperglycemia leads to overexpression of SGLT2 cotransporters, which causes an increase in glucose absorption in PCT. The tubuloglomerular feedback is activated as a result of reduced sodium delivery to the juxtaglomerular complex, which induces dilatation of afferent arterioles, contributing to an elevation in intraglomerular pressure and an increase in glomerular filtration rate above average values-a distinctive feature of kidney disease in diabetes [26]. The gliflozins undo this process. This leads to a decrease in intraglomerular pressure and an improvement in hyperfiltration [26,31]. When used with beta-blockers or calcium antagonists, gliflozins exhibit more significant antihypertensive effects than thiazide diuretics [32,33]. In patients with HF, inflammation is an essential contributor to the severity; elevated proinflammatory biomarkers correlate with the disease severity [34,35]. SGLT2-Is bring about a state of ‘fasting mimicry’ by enhancing urinary glucose loss. This, in succession, activates the enzymes possessing antioxidant and anti-inflammatory properties, mainly SIRT1 and AMPK, which improve cardiac function [36,37]. The anti-inflammatory properties of dapagliflozin have been shown to reduce the synthesis of collagen and thereby inhibit fibrosis and scarring in rats after myocardial infarction (MI) [38]. Hess et al. [39] noted that empagliflozin administration resulted in a decrease in proinflammatory M1 cells and an increase in anti-inflammatory M2 cells.

Noteworthy improvements were found in the flow-mediated dilation of the brachial artery when investigating the use of dapagliflozin in patients with poorly controlled glycated hemoglobin in the dapagliflozin effectiveness on vascular endothelial function and glycemic control (DEFENCE) study [40]. However, the same effect is observed with other classes of diabetes medication, which gives the idea that the association between SGLT2-Is and improved endothelial function may be indirect rather than direct [41]. Gliflozins reduce the occurrence of the early formation of fatty deposits in the arteries, contributing to better vascular function. Furthermore, gliflozins also reduce activation of the endothelium and vascular resistance [42-45]. In Zucker fatty and spontaneously hypertensive (ZSF1) rats with metabolic syndrome and insulin resistance, empagliflozin decreased the expression of atherothrombotic markers and improved endothelial function [46]. By promoting urinary glucose excretion in a mouse with T2DM, empagliflozin reduced the stiffness of the aorta and improved endothelial dysfunction [47]. In a comparative experiment conducted by Tahara et al. [48] on a T2DM mouse, the positive effects of six SGLT2-Is (luseogliflozin, ipragliflozin, tofogliflozin, empagliflozin, canagliflozin, and dapagliflozin) were compared, and it was noted that all six drugs were shown to improve endothelial dysfunction, which is a class effect of these agents. Gaspari et al. [43] observed that dapagliflozin lessened the activation of the endothelium and promoted significant vascular relaxation independent of the endothelium.

**Figure 1:**
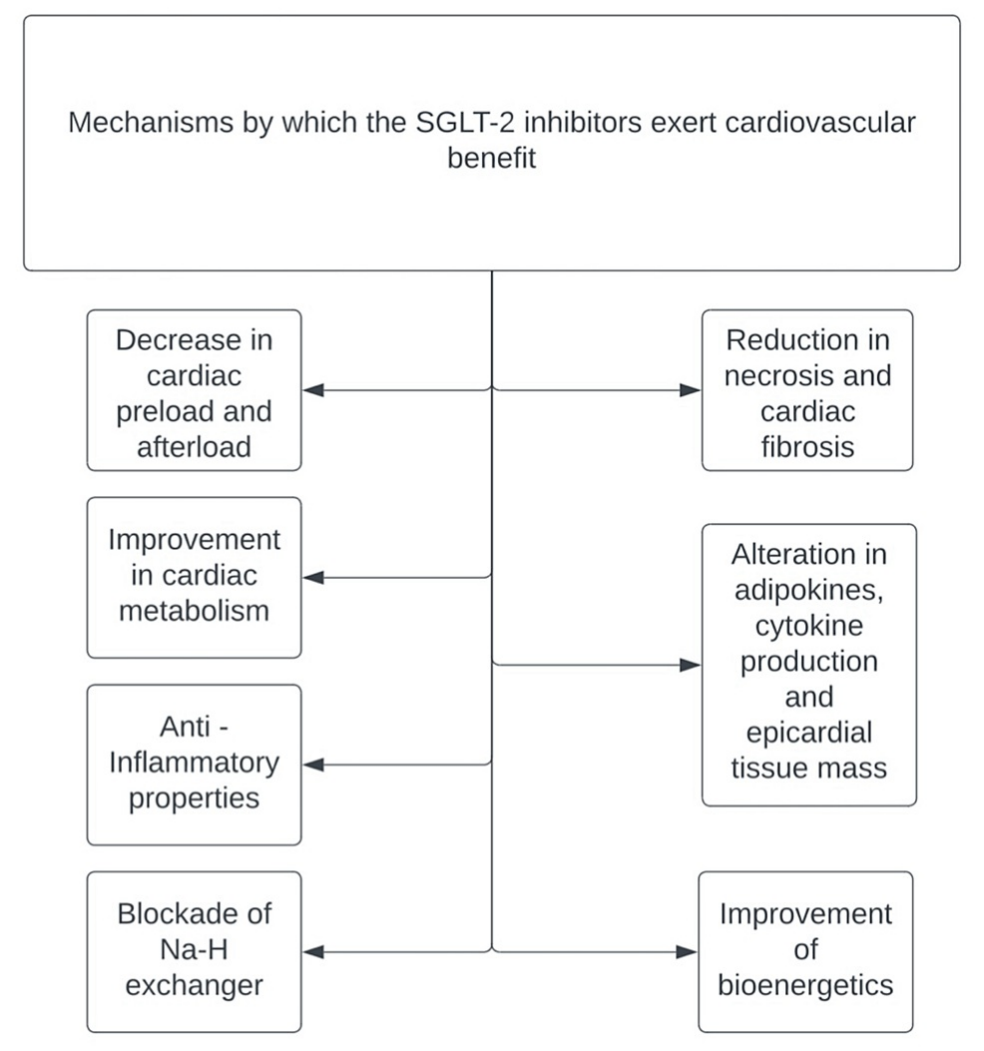
Proposed mechanisms to describe the effect of SGLT2-Is on cardiovascular health SGLT2-Is: sodium-glucose cotransporter-2 inhibitors [12-16]; Na-H exchanger: sodium hydrogen exchanger Image credits: Sweatha Mani

Overactivity of the cardiac Na-H exchanger is linked to heightened chances of HF. SGLT2-Is block these channels [10], subsequently enhancing the responsiveness to diuretics and endogenous natriuretic peptides and reducing cardiac fibrosis, hypertrophy, and remodeling [49]. SGLT2-Is improve myocardial energy efficiency by facilitating the body's production of ketones, a more energy-efficient substrate than fatty acids or glucose [50]. Although gliflozins are SGLT2-Is, inhibition of SGLT1, which is found in the heart, is observed with canagliflozin. This results in lower absorption of sodium and glucose by the heart and thereafter leads to a decrease in reactive oxygen species (ROS) production triggered by increased sugar levels [51]. Treatment with clinically relevant doses of canagliflozin [52], ipragliflozin, and empagliflozin [53] inhibited the growth of vascular smooth muscles and neointimal proliferation. On top of this, empagliflozin also improved coronary microvascular and contractile function [54]. SGLT2-Is reduces the subunits of NADPH oxidase, such as NOX 1, NOX2, NOX4, and p22phox [55-58]. The activation of NLRP3 inflammasome plays a vital role in inflammation by releasing interleukins [59], thereby contributing to the pathogenesis of atherosclerosis and HF [60-62]. In T2DM and cardiovascular disease patients, Kim et al. [63] found that empagliflozin inhibited the NLRP3 inflammasome regardless of glycemic control. The same effect was observed with dapagliflozin in aortic tissues, reversing the formation of atherosclerosis [64]. The proposed mechanisms are explained in Figure 1. 

Cardiovascular outcome trials

SGLT2-Is have been shown to remarkably reduce the risk of major adverse cardiovascular events (MACE), HHF, MI, stroke, and HF [65]. These findings are from the real-world meta-analysis of 14 trials where 3,157,259 participants were enrolled with T2DM, of which 11.2%~33.4% of the study population had a previous cardiovascular event. The Dapagliflozin Effect on Cardiovascular Events-Thrombolysis in Myocardial Infarction 58 (DECLARE-TIMI 58) trial focusing on dapagliflozin [66], the CANagliflozin cardioVascular Assessment Study (CANVAS) trial on canagliflozin [29], and the Empagliflozin Cardiovascular Outcome Event Trial in Type 2 Diabetes Mellitus Patients-Removing Excess Glucose (EMPA-REG OUTCOME) trial on empagliflozin [67] validated the cardiovascular benefits of SGLT2-Is mentioned above. The DECLARE-TIMI 58 trial was conducted to observe whether dapagliflozin accomplished cardiac and renal safety in participants with or prone to atherosclerotic cardiac disease [66]. The CANVAS Program, comprising two sister trials (CANVAS and CANVAS-R), evaluated canagliflozin’s cardiovascular well-being and effectiveness [29]. The EMPA-REG OUTCOME trial validated the effects of empagliflozin on cardiovascular morbidity and death in participants with T2DM at heightened risk for cardiovascular events who were receiving standard care [67].

The DECLARE-TIMI 58 trial conducted by Wiviott et al. [66] included 17,160 diabetic participants, multi-nationally comprising 6,974 patients with established cardiovascular disease and 10,186 patients with multiple risk factors (men aged 55 years and above and women aged 60 years and above with hypertension, dyslipidemia, and tobacco usage). The participants were given either placebo or dapagliflozin and were monitored for an average of 50.4 months. Neal et al. [29] conducted the CANVAS program. The program included 10,142 participants with T2DM and a heightened chance of cardiac and vascular disease. The participants were ambiguously allotted; either canagliflozin or a placebo was given for a mean duration of 43.3 months. Zinman et al. [67] conducted the EMPA-REG OUTCOME trial, a randomized placebo-controlled trial published in 2015. The study included a total of 7,020 participants with T2DM with established cardiovascular disease, mainly stable coronary artery disease. The participants received 10 mg or 25 mg of empagliflozin or placebo and followed up for a median of 3.1 years [67]. The findings are shown in Table 1.

**Table 1:** Cardiovascular outcome trials MACE: major adverse cardiovascular event, a combination of ‘death from cardiovascular causes, nonfatal myocardial infarction (MI), and nonfatal stroke; HHF: hospitalization due to heart failure; renal composite: a steady decline of 40% or higher in estimated glomerular filtration rate (eGFR), the occurrence of end-stage kidney failure, or fatal outcomes from kidney and heart-related causes.

References	Design	Total Participants	Population	Assessed Primary outcome	Assessed Secondary outcome	Conclusion
Wiviott et al. [66]	Randomized controlled trial with dapagliflozin	17,160 with type 2 diabetes	6,974 with established cardiovascular disease and 10,186 with multiple risk factors	MACE, cardiovascular death, HHF	Renal composite, death from any cause	No change in MACE and secondary outcomes, Decrease in CV death and HHF
Neal et al. [29]	Randomized controlled trial with canagliflozin	10,142 with type 2 diabetes	Elevated risk of cardiovascular disease	MACE	Death from any cause, albuminuria, HHF	Decreased rates of MACE and albuminuria
Zinman et al. [67]	Randomized controlled trial with empagliflozin	7,020 with type 2 diabetes	Increased cardiovascular risk	MACE	Hospitalization for unstable angina	Decreased rates of death from cardiovascular causes, HHF, and death from any cause.

Effect of SGLT2-is on MACE, HHF, and renal outcomes

A MACE is the composite of deaths from cardiovascular causes, nonfatal MI, and nonfatal stroke. It is observed from the CANVAS program that markedly fewer patients who received canagliflozin had MACE when compared to the placebo group [29]. Whereas in the EMPA-REG OUTCOME TRIAL, empagliflozin resulted in a significantly lower risk of death from cardiovascular causes, with no notable differences in the rates of MI or stroke [67]. Regarding the efficacy of dapagliflozin in the DECLARE TIMI-58 trial, it has been shown to reduce the rates of HHF compared to the placebo. Additionally, this efficacy is the same in the subset of patients with established atherosclerotic cardiovascular disease and those with multiple risk factors [66]. Compared with the placebo, in the EMPA-REG OUTCOME trial, empagliflozin significantly reduced the HHF [67]. The renal outcome, which was focused on in the DECLARE TIMI-58 trial, is the combination of a steady decrease in glomerular filtration rate and new end-stage kidney failure or mortality due to renovascular or coronary causes. It was observed that dapagliflozin reduced the composite renal outcome in the trial population [66]. Canagliflozin reduced the progression of albuminuria more than those allocated with placebo, a noteworthy finding in the CANVAS trial [29].

Challenges of SGLT2-Is

In the DECLARE TIMI 48 trial, one of the serious adverse effects noted in the dapagliflozin group, although rare, was the upsurge in genital infections in both males and females, which led to the discontinuation of the trial. Other observed adverse effects were renal failure, which was acute in onset (acute renal failure (ARF)), a significant fall in sugar levels, urothelial carcinoma, and Fournier’s gangrene [66]. In the CANVAS program, an increased risk of amputation at the level of the toes, feet, or legs concerning canagliflozin was found. In participants who had an amputation previously or had arterial insufficiency in their legs, these findings were increasingly noted. Other reported effects were infections of male and female genitalia, volume depletion and diuresis, and an increased rate of fractures. A small number of participants were reported to have diabetic ketoacidosis (DKA) [29]. In the EMPA-REG OUTCOME, a higher percentage of patients reported genital infections in the empagliflozin group. There was no change in the percentage of patients with established hypoglycemia, ARF, DKA, thromboembolism, fracture, or depletion of volume. Urosepsis was noted to be increased in the empagliflozin group (0.4%) as compared to the placebo (0.1%) [67].

Limitations

This review article only includes studies from PubMed and Google Scholar. The pathogenesis and treatment of HF involve a multifactorial aspect, while this study only focused on SGLT2-Is.

## Conclusions

In recent times, corroboration from multiple trials has demonstrated the unanticipated but beneficial effects of SGLT2-Is. The initiation of evidence-based treatment should not be deferred by clinical inertia. The commencement of effective treatment modalities early in the disease course can significantly reduce morbidity and mortality worldwide. As discussed in the large cardiovascular outcome trials, SGLT2-Is should be considered foundational therapy in patients with HF and with standard HF drugs such as angiotensin-converting enzyme inhibitors, angiotensin receptor blockers, diuretics, and beta-blockers. In the present review, we summarized data supporting the initiation of gliflozins in HF patients. These drugs embody most of the typical characteristics of an HF medication: no need for dosage adjustments, early reported beneficial effects on clinical events, improved quality of life, and a proven safety and tolerability profile with minimum adverse effects. Crucially, it still needs to be clarified whether the benefit of gliflozins extends over a long period and if the therapy can forestall damage to the heart and kidneys. So, it is imperative that further studies be conducted in the future to understand the benefits and multiple routes by which the SGLT2-Is act in detail.
